# Improving conscientiousness through a smartphone app and telecoaching intervention: insights from multiple sclerosis and healthy aging samples

**DOI:** 10.1007/s00415-025-13320-9

**Published:** 2025-08-15

**Authors:** Michael Jaworski, Jacob Balconi, Celeste Santivasci, Margaret Youngs, Morgan Woodbeck, Nathan Zak, Sara Cruz, Bianca Weinstock-Guttman, Ralph H. B. Benedict

**Affiliations:** 1 Department of Neurology, Jacobs School of Medicine and Biomedical Sciences, Jacobs Multiple Sclerosis Center, University at Buffalo, The State University of New York, Buffalo, NY USA; 2 Buffalo Neuroimaging Analysis Center, Department of Neurology, Jacobs School of Medicine and Biomedical Sciences, University at Buffalo, The State University of New York, Buffalo, NY USA; 3 Department of Psychology, Louisiana State University, Baton Rouge, LA USA; 4 Department of Psychology, Pennsylvania State University, University Park, PA USA

**Keywords:** Conscientiousness, Personality intervention, Telecoaching, Multiple sclerosis, Health aging, Functional impairment

## Abstract

Lower Conscientiousness is associated with cognitive decline, unemployment, and poorer health outcomes in both neurological and aging populations. This study evaluated a smartphone-based intervention to enhance Conscientiousness in people with multiple sclerosis (PwMS) and healthy aging (HA) adults. Initially, 38 PwMS and 22 HA participants completed baseline assessments (cognitive, personality, depression, and anxiety measures). Attrition and disease factors left 24 PwMS and 20 HA adults at the 12-week follow-up. Participants randomly assigned to the intervention arm received the “Conscientiousness Coach” app, which combined value identification, SMART goal development, smartphone-based tracking, and scheduled telehealth coaching sessions, whereas controls were wait-listed. ANCOVA for baseline scores showed a significant treatment effect on NEO-FFI Conscientiousness (p = 0.015) that remained after substituting age for diagnosis (p = 0.029). A lack of significant effects for group (PwMS vs. HA) and age indicated that treatment impact was consistent across both samples. Within the intervention group, paired-sample t-tests revealed large gains in the Conscientiousness domain (*d* = 0.93, p < 0.001) and moderate improvements in the Orderliness (*d* = 0.67, p = 0.007) and Dependability (*d* = 0.52, p = 0.030) facets, with no change in wait-list controls. No treatment effects emerged for Neuroticism, depression, or anxiety, underscoring the trait-specific nature of the intervention. Results suggest that targeted digital interventions can favorably impact personality and enhance quality of life. Future study will examine use of AI to replace the behavioral coaching component of this intervention and its long-term impact on personality.

## Introduction

The Five-Factor Model (also known as the Big Five) of Personality is widely accepted internationally as a gold standard conceptualization in the psychological and medical literatures [1–6]. Conscientiousness, one of the traits in the Five Factor Model, is the propensity to be achievement striving, dependable, organized, and persistent [7, 8]. There is a well-established and growing literature showing that Conscientiousness favorably impacts behavioral adaptation. This trait is strongly correlated with a range of positive outcomes, including enhanced academic performance and greater career success [9]. Conscientiousness is low in people with neurological disorders such as Parkinson’s disease [10–12], aging related mild cognitive impairment [13] and Alzheimer’s disease [14–16]. In addition, higher Conscientiousness is associated with favorable health outcomes in diabetes, and cardiovascular disease [17, 18]. In people with diabetes, this trait is associated with greater control of blood glucose levels [19].

Multiple sclerosis (MS) is an immune-mediated neurodegenerative disease of the central nervous system, characterized by demyelinating lesions. The lesions are found throughout the brain and spinal cord, causing neuronal loss and the accumulation of neurologic impairments [20]. People with MS (PwMS) experience a wide range of symptoms, including sensory and motor impairments, gait and balance difficulties, spasticity, visual disturbances, and cognitive impairment [21]. These symptoms can vary widely in their severity and impact on daily functioning, career, relationships, social obligations, and overall quality of life (QoL) [22–24]. Cognitive impairment [25] is particularly relevant for maintaining employment in MS [26]. In PwMS, higher trait conscientiousness at baseline predicts slower decline in cognitive functioning over time [27] and reduced progression of brain atrophy [28]. Patients low in Conscientiousness are also at higher risk for unemployment [29]. We [30], and others [31], find that trait Conscientiousness significantly influences response to cognitive training.

Conscientiousness is associated with functional and economic independence in aging [32, 33], activities of daily living, independent of medical morbidity [34]. In longitudinal studies of geriatric primary care patients Conscientiousness and also Neuroticism predict greater illness burden, functional impairment [35] and nursing home placement [36]. There is burgeoning interest in how these traits influence modifiable risk factors for dementia due to Alzheimer’s disease (AD). Cross-sectionally, AD patients have higher Neuroticism and lower Conscientiousness than healthy persons [37, 38]. To some extent this reflects disease-related neurodegeneration, but meta-analytic estimates show that premorbid low Conscientiousness prospectively predicts incidence and progression of dementia [13]. Similar effects are found in the AD prodromal state amnestic mild cognitive impairment (MCI) [39].

How Conscientiousness (and to a lesser degree Neuroticism) impact neurological disease is not clear. Some research indicates that these traits have stronger association with the diagnosis of dementia than the underlying cerebral pathology [40], suggesting an effect similar to the concepts of cognitive reserve or neuronal resilience. Also, there may be direct effects on health behavior that carries over to biomarkers such as exercise tolerance, blood pressure, smoking, and alike [41].

Although traditionally considered stable throughout adulthood [42], recent research challenges this notion [43, 44]. Online behavior-change interventions have been demonstrated to produce modest to moderate increases in trait Conscientiousness among healthy young adults [45, 46]. However, to our knowledge, no remote digital intervention has specifically targeted Conscientiousness as a primary outcome in clinical, neurocognitive, or aging populations, which are highly susceptible to the detrimental effects of maladaptive personality traits.

To address this gap, we conducted a 12‑week randomized controlled trial of the “Conscientiousness Coach,” combining the smartphone app with telehealth coaching sessions. We tested this protocol in two at-risk populations: PwMS and community-dwelling older adults (age ≥ 60 years). Both groups, one defined by chronic neurological disease and the other by age-related vulnerability, are known to experience cognitive decline and functional impairments linked to low Conscientiousness. In this study, we explored the possibility of enhancing trait Conscientiousness. Given that low Conscientiousness has been associated with cognitive and functional impairments in aging and neurological disorders, while high Conscientiousness provides protective benefits, we hypothesized that a targeted intervention could potentially boost the trait across distinct risk profiles. Herein, we describe our recent attempt to modify Conscientiousness in both a clinical neurocognitive sample and an at‑risk older adult population.

## Methods

This study was approved by the University at Buffalo institutional review board. All participants provided informed consent and were compensated for their time and effort.

### Participants

**Fig. 1:**
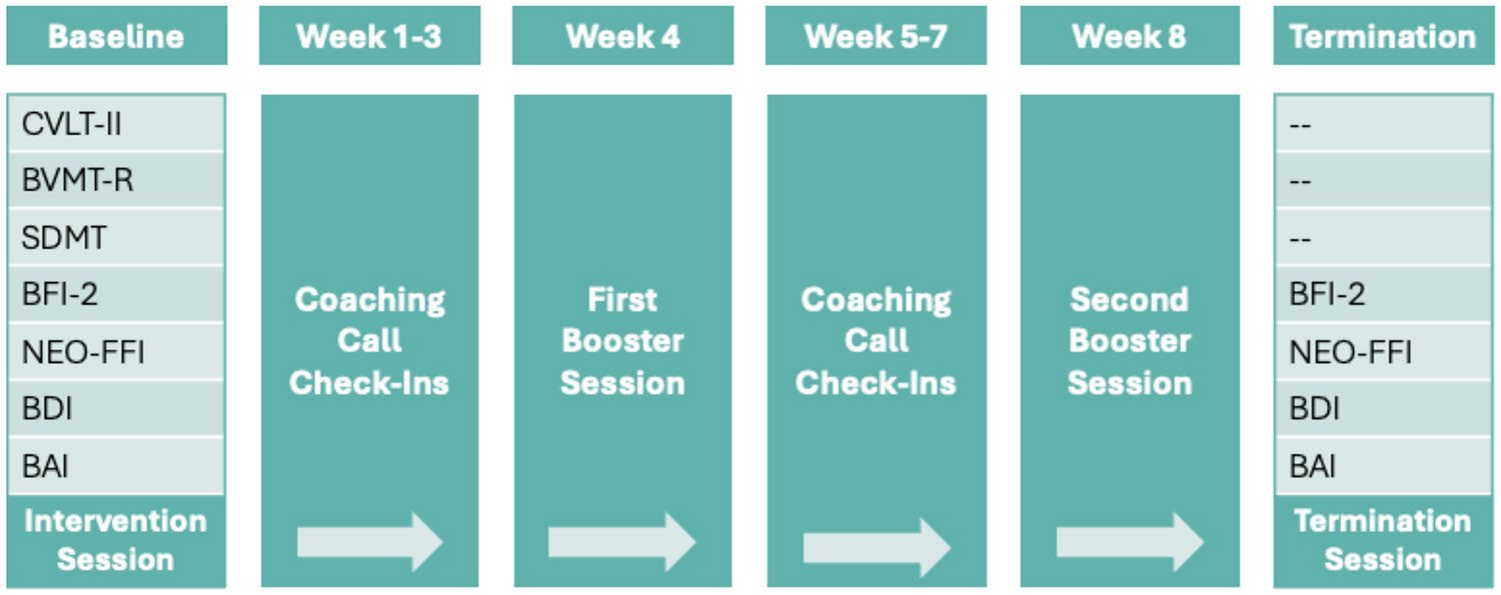
Study Timeline. *Note.* Treatment group participants underwent a 12-week intervention initially undergoing a baseline neuropsychological evaluation and intervention totaling approximately 2 h. Baseline assessments (CVLT-2, BVMT-R, SDMT, BFI-2, NEO-FFI, BDI, BAI) were conducted prior to the intervention. The intervention included education on conscientiousness, values evaluation, and development of SMART goals. Participants were trained to use the conscientiousness coach app and received weekly check-ins (C. Calls) to address technical issues and review progress. Booster sessions were conducted during weeks 4 and 8 to reinforce conscientiousness concepts, adapt goals, address obstacles, and recognize achievements. Termination assessments included the same neuropsychological measures as baseline, along with a closing session encouraging continued app use. Waitlist controls underwent the same assessments but did not receive the intervention

This study recruited two distinct samples: [a] 38 individuals with clinically definite multiple sclerosis [47] and [b] 22 healthy aging (HA) individuals. Inclusion criteria for both groups were no self-reported medical, psychiatric, or developmental disorders that affect cognition, no prescription of antipsychotic, narcotic, or anticonvulsant medication, no self-reported drug or alcohol use disorder, and for PwMS, no clinical relapse during study participation. Access to a smartphone was required for participation. For the MS group, we enrolled those with a NEO Five Factor Inventory (NEOFFI) [48] Conscientiousness sex-corrected T-score of 45 or less, regardless of age. This T-score threshold was not required for HA participants, who were aged 60 and older and recruited from the University at Buffalo’s Participate in Research Portal. The HA group was not conceptualized as a control condition but rather as an independent, age-defined risk cohort, allowing us to examine cross-population generalizability of the intervention’s effects. Once recruited, participants were randomly assigned to either an intervention or waitlist control arm. Both were assessed using the same psychological measures at baseline and follow-up, as shown in Fig. 1. Of the 38 PwMS and 22 HA participants, 12 PwMS (7 control, 5 intervention) and 2 HA participants (1 control and 1 intervention) discontinued study participation. Additionally, 2 PwMS in the intervention group were removed after experiencing exclusion criteria during study participation (i.e., 1 major depressive episode and 1 clinical MS relapse).

### Conscientiousness coach intervention

The intervention commenced with a 60-min in person coaching session wherein participants were taught how to use the app. Using expectancy value theory [49, 50], participants were educated about the trait Conscientiousness, and the importance of behavior being guided core life values. Values were defined as the core principles and ideals that are central to shaping one’s thoughts, choices, and behaviors. After some time developing and refining core values, participants selected some values that would be entered into the app. For each value, goals were developed for each selected value. Participants set goals that were specific, measurable, achievable, realistic, and time-bound (SMART) [51, 52]. For instance, instead of setting a vague goal such as “be more spiritual,” participants defined specific goals, such as dedicating 5 min every night on weekdays to reading a religious text or meditating. By guiding participants to work methodically within intrinsically motivating goals, scheduled to a particular time on a calendar or on a “to do” list. Throughout the intervention the app tracked goal achievement and coaching contacts by phone helped to integrate goal achievement with the idea of value actualization. Scheduled telehealth check-in sessions lasting 30 min were scheduled for weeks 3, 5–7, and 9–11. Two 60-min telehealth coaching booster sessions were scheduled for weeks 4 and 8. Participants discussed challenges and obstacles, solutions to enhance productivity, and clarification of values. Participants had the option of adding or discontinuing activity in specific values. Participants were invited to reflect on their personal growth and life changes, reaffirming their progress. At week 12, participants had a final meeting with the coach for a debriefing session.

The study utilized an enhanced version of the “Conscientiousness Coach” app [53], building upon the initial iteration to effectively instruct individuals. The app incorporates notifications that serve as reminders aligned with values and goals, reinforcing essential aspects of conscientiousness, including dependability and self-accountability. On the home page, values are prominently showcased and represented by icons, each symbolizing the corresponding value. These icons form a visually appealing “value-circle” that signifies the participants’ core ideals and beliefs. The app includes specific reminders that prompt participants to regularly reflect on their value circle and contemplate how their behaviors may impact these values. By selecting an icon within the circle, participants gain access to a dedicated page tailored to the specific value. On value pages, participants have the flexibility to input or modify their subordinate goals, customize reminders, and diligently track their goal progress, including streaks of consistent achievement.

### Assessment

The assessments were carried out by a research assistant blinded to group assignment. Similarly, the overseeing coach responsible for the intervention was blinded to all pre- and post-intervention outcomes. The assessment included the California Verbal Learning Test (CVLT-II) [54], a measure of verbal learning, the Brief Visuospatial Memory Test Revised (BVMT-R) [55], and the Symbol Digit Modalities Test (SDMT) [56], a measure of processing speed where the participant has 90 s to match symbols to numbers. Questionnaires included the NEOFFI [48] and the Big Five Inventory 2nd Edition (BFI-2) [57], 60-item self-report questionnaires assessing the big five personality traits (Openness/Open-Mindedness, Conscientiousness, Extraversion, Agreeableness, and Neuroticism/Negative Emotionality); the Beck Depression Inventory 2nd Edition (BDI-II) [58], a measure of depressive symptoms consisting of 21 items; and the Beck Anxiety Inventory (BAI) [59], a 21-item measure of anxiety symptoms. Questionnaires were readministered at the 12-week follow-up.

### Statistical analyses

All variables were assessed for normality, and all outcomes approximate a gaussian distribution except for positive skew for the BDI2 and BAI. Independent samples t-tests were applied to baseline measures, comparing the MS and HA samples. The cognitive performance measures were normalized by age, sex and education by previously published regression-based norms [60, 61].

As this was a preliminary and exploratory study, we did not select a single primary outcome but considered two co-primary outcomes: the NEO-FFI Conscientiousness domain and the BFI-2 Conscientiousness domain. All other measures (e.g., NEO-FFI Neuroticism, BFI-2 Negative Emotionality, BDI-II, BAI) were treated as secondary or exploratory outcomes to assess the specificity of the intervention’s effects.

To investigate the intervention’s influence across all study participants, we first conducted analysis of covariance (ANCOVA) where the post-treatment outcome was compared between treated and control arms, and the pre-treatment baseline value was included as a covariate. We included treatment (active versus control) and diagnosis (MS vs. HA) as covariates. Additionally, to assess whether treatment effects differed by age, we utilized a second model replacing the diagnosis factor with age. This was done for the following measures: NEOFFI Conscientiousness, BFI-2 Conscientiousness, NEOFFI Neuroticism, BFI-2 Negative Emotionality, BDI-2 and BAI. If there were no significant effects, a priori, there would be no further interrogation of the data. If the treatment variable was significant favoring the active group, we planned to conduct paired-samples t-tests on the subscales to determine whether a specific component of Conscientiousness was more relevant than others. Significant findings on the NEO-FFI or BFI-2 measures were followed by analyses of the domain scores.

## Results

### Baseline data

As shown in Table 1, PwMS were younger than HA participants (p < 0.001). As expected, PwMS had higher levels of anxiety (BAI) (p = 0.010) and depression (BDI-2) (p = 0.026). They were also more cognitively impaired for their age, sex, and education. This was most apparent on the SDMT where the MS mean Z-score was −0.79 and the HA mean was 0.70 (p = 0.001). In addition, Neuroticism was elevated, and Conscientiousness was lower among PwMS, on both the NEOFFI and BFI-2 personality measures (all p < 0.05). This is expected, as the MS sample was recruited with an entrance criterion of NEOFFI Conscientiousness T-score < 45.

**Table 1:** MS and HA Baseline Comparisons

Measure	PwMS (n = 24)		HA (n = 20)		p-value
	M	SD	M	SD	
Age	51.3	12.6	68.5	5.6	< 0.001
Sex (% Female)	66.7	–	75.0	–	0.546
Education	14.7	2.3	15.5	2.4	0.258
CVLT-2 TL (z)	− 0.5	1.1	– 0.5	0.9	0.928
CVLT-2 DR (z)	− 0.2	1.2	0.3	1.0	0.186
BVMT-R TL (z)	0.1	1.3	0.9	1.0	0.026
BVMT-R DR (z)	0.1	1.4	1.1	1.2	0.014
SDMT (z)	− 0.8	1.7	0.7	1.0	0.001
NEOFFI C	34.9	11.2	50.6	10.6	< 0.001
BFI-2 C	43.8	9.6	59.6	8.8	< 0.001
NEO-FFI N	54.0	11.3	45.6	9.8	0.012
BFI-2 N	51.9	11.8	42.8	10.9	0.012
BAI	13.0	9.8	6.7	4.4	0.010
BDI-II	14.9	10.4	8.6	6.8	0.026

### Effects of intervention

The ANCOVA comparing treatment arms on post-treatment NEOFFI Conscientiousness, showed a significant effect for treatment (p = 0.015) but the diagnosis effect was not significant (p = 0.364). This indicates that those undergoing the “Conscientiousness Coach” app intervention changed to a greater degree than those in the wait-list control arm, regardless of diagnosis. Replacing the diagnosis factor with age did not alter the significance of the treatment effect (p = 0.029), and the age effect itself was not significant (p = 0.128). Similar results were obtained for the BFI-2 measure of Conscientiousness. The treatment effect was approaching significance when controlling diagnosis (p = 0.053), as well as age (p = 0.057). The effects of diagnosis and age were not significant (p = 0.458; *p* = 0.835, respectively).

**Fig. 2:**
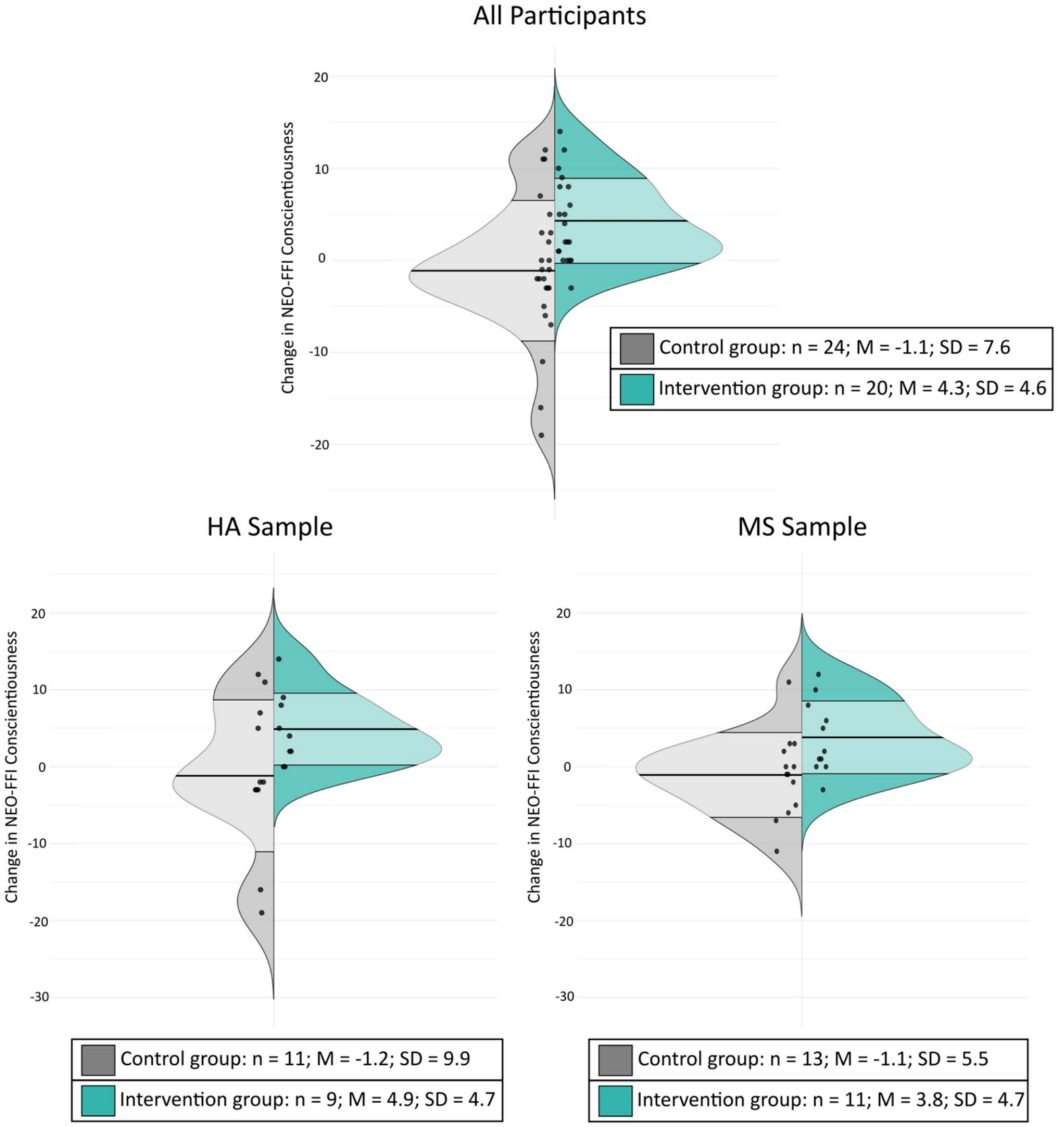
Change in Conscientiousness T-Scores by Group *Note.* Split violin plots showing change in NEO-FFI Conscientiousness T-score (follow-up – baseline). In each plot, the left half represents the distribution of change in the control group, while the right half shows the intervention group’s distribution. The central line in each distribution represents the mean, with shaded regions representing mean ± 1 standard deviation, *n* number of participants in the group; *M* mean; *SD* standard deviation; *HA* healthy aging; *MS* multiple sclerosis

**Fig. 3:**
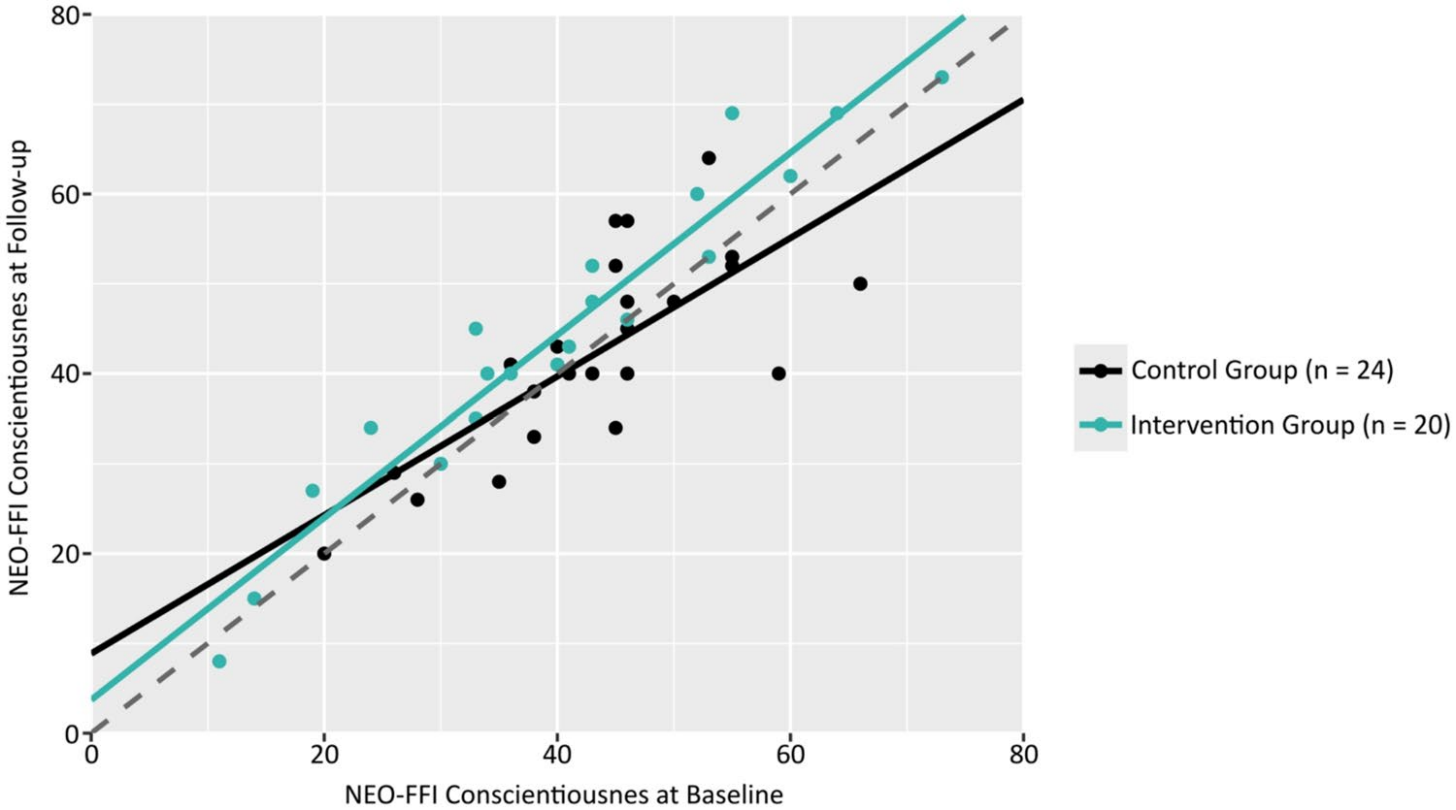
Baseline vs. Follow-Up Conscientiousness by Group *Note.* Scatterplot showing the relationship between NEO-FFI Conscientiousness ratings at baseline and follow-up, by treatment group. The solid lines for each group represent the linear relationship between baseline and follow-up ratings. The dashed regression line represents the function y = x, n = number of participants in the group

The effects of treatment can be appreciated in violin plots of change scores shown in Fig. 2. Here, there is plainly visible a higher frequency of improvement in treated participants, and this difference is equally evident in both samples, demonstrating the null diagnosis effect in the ANCOVA model Fig. 3

For Neuroticism, there were no treatment effects for either the BFI-2 or NEOFFI outcomes. There was a borderline trend for treatment on the NEOFFI when controlling for diagnosis (p = 0.108), but as this may be a chance finding we did not explore the analysis. As above, the diagnosis and age effects were not significant (p = 0.846; p = 0.295, respectively).

The ANCOVA models for anxiety and depression symptoms were not significant. The BAI model did show a borderline trend for a treatment effect favoring the intervention arms of questionable significance (p = 0.122).

For NEOFFI Conscientiousness, univariate, within group comparisons were examined using paired sample t-tests, for the domain score as well as the facet scores (Table 2). The intervention group showed significant gains in the domain score (*d* = 0.93, p < 0.001), and facets Orderliness (*d* = 0.67, p = 0.007) and Dependability (*d* = 0.52, p = 0.030). There was no evidence of improvement in the control group arms.

**Table 2:** Baseline to follow-up comparisons by group

Outcome	Intervention Group (n = 20)						Waitlist Control Group (n = 24)					
	Baseline		Follow-up		*d*	p-value	Baseline		Follow-up		*d*	p-value
	M	SD	Mean	SD			M	SD	M	SD		
NEO-FFI C	40.20	16.40	44.50	17.30	0.93	< 0.001	43.50	10.40	42.40	10.80	−0.15	0.478
Orderliness	40.90	13.30	44.50	15.50	0.67	0.007	43.00	9.80	42.50	9.00	−0.08	0.714
Goal Striving	48.50	11.20	50.60	11.30	0.29	0.214	50.60	10.00	51.30	10.10	0.08	0.700
Dependability	43.00	15.50	46.90	15.90	0.52	0.030	46.40	12.80	43.50	12.90	−0.46	0.035

## Discussion

Recognizing that trait Conscientiousness is a protective factor for cognitive decline and other clinical parameters in neurological disease we set out to test a novel intervention to enhance this important personality trait. This study is the first to integrate a smartphone‑based “Conscientiousness Coach” app with telecoaching to modify Conscientiousness in PwMS and healthy older adults. By demonstrating the feasibility and efficacy of this digital‑telehealth model, we lay the groundwork for future trials assessing its potential to reduce MS‑related disability and delay age‑related cognitive decline.

We developed a smartphone app that functions as the primary tool for this behavioral intervention. The statistical analyses show that participants utilizing the “Conscientiousness Coach” showed significant gains in Conscientiousness as measured by the NEOFFI. Additionally, by including the NEO‑FFI Conscientiousness subfacets, rather than just the trait as a monolith, our intervention yielded significant improvements in subfacets Orderliness and Dependability, offering more nuanced insights than prior global trait studies. These results suggest that personality traits, traditionally considered immutable, can be modified through targeted interventions, offering promising avenues for improving both psychological and emotional well-being in PwMS and aging individuals.

We are just at the beginning stages of this research, as only recently has the concept of personality trait change appeared in the literature. We do not know the mechanism of action, nor its long-term impact on quality of life or activities of daily living. The intervention emphasizes the importance of aligning behaviors with personal “core” life values. The “Conscientiousness Coach” intervention may foster an increased sense of autonomy and relevance, and a commitment to achievement. Enhancing Conscientiousness may lead to improved goal setting, better adherence to treatment plans, and engagement with positive health behaviors. Longer studies are needed to determine if these behavioral changes related to Conscientiousness are maintained over months or years.

The use of a companion smartphone app is important to reinforce the intervention’s principles. The app was the second iteration designed to more effectively instruct and support individuals in Conscientious behaviors [53]. Developed using the React Native platform, the application was simultaneously designed for both iOS and android devices, ensuring accessibility across a wide range of smartphones. This cross-platform functionality allowed for a seamless and standardized experience regardless of device, making the intervention more widely scalable. To further promote Conscientious behaviors, the app provides the user custom notifications that act as reminders of both personal values and goals, encouraging individuals to reflect on their commitments and how their daily behaviors align with their “value circle.” Coupled with telecoaching, this structured yet flexible design demonstrates a viable digital‑health model for self-directed behavior change.

The application is undergoing further development. In this study, it lacked built-in motivational features commonly found in modern “habit trackers,” such as streak tracking (of goals), or other adaptive reinforcement mechanisms. Additionally, self-reflection options were limited. Users could set values, goals and reminders, but the application did not prompt them to assess whether a goal was truly SMART or whether a value held intrinsic significance. The intervention relied on the human coach interface for these components of the intervention. Furthermore, the absence of professional UI/UX design expertise led to some navigation challenges. For instance, daily goals were not displayed on the home screen but instead required users to access individual value pages, adding unnecessary steps to goal tracking. Finally, in an era of rapidly advancing artificial intelligence, the app did not incorporate AI-driven functionalities that could have enhanced real-time personalization. AI-based features could dynamically adjust reminders, suggest refinements to values or goals, and provide immediate feedback on whether a goal aligns with SMART criteria.

The findings of this study have significant implications for clinicians, particularly in the management of chronic conditions like MS. Incorporating personality enhancement strategies into treatment plans could improve adherence (e.g., medication), engagement in rehabilitation activities, and overall quality of life. Healthcare professionals may consider integrating a form of this intervention into their recommendations to promote conscientious behaviors that support disease management. For HA individuals, enhancing conscientiousness may contribute to healthier aging by fostering behaviors that protect against cognitive decline and promote physical health. When one considers the many behaviors that are targeted by healthy aging advocates, the question emerges of what sort of person is organized enough to engage in these behaviors. Personality is a therapeutic target that may be construed as a latent variable to be considered in prevention strategies. Given that conscientiousness has been linked to better health outcomes (e.g., reduced risk of cardiovascular disease, better glycemic control in diabetes, increased longevity, and lower levels of amyloid-beta deposition), interventions targeting this trait could be valuable components of preventive health strategies for older adults.

The NEOFFI Conscientiousness facet scores were analyzed and there seemed to be a more significant impact on dependability than goal achievement. Sutin and colleagues [62] have found that Responsibility is closely linked to cognitive outcomes in healthy aging individuals and may be an important mediator of the positive effects of Conscientiousness as a whole. The authors note that this facet of Conscientiousness emphasizes service to others, the interpersonal component of Conscientiousness, much like the Dependability facet reported on here. We may find that this aspect, increasing meaningful, yet social behaviors, is a key component to the positive impact of increasing Conscientiousness.

Despite the promising results, this study has limitations that should be acknowledged. The sample size was small, which limits generalizability and statistical power. The reliance on self-report measures for personality traits and emotional well-being may introduce bias, although validated measures were used. A notable limitation was the higher dropout rate among PwMS compared to HA participants. Notably, individuals in the MS group who were lost to follow-up had lower Conscientiousness scores compared to those who completed the study, with this difference approaching statistical significance (mean T-scores: 27.7 vs. 35.2; *p* = 0.065). These MS groups did not significantly differ on any other Table 1 measure (e.g., demographics, cognition, personality) or in disease course (i.e., Relapsing–Remitting vs. Secondary Progressive). It may be the case that very low Conscientiousness persons lack the stability and organization to participate in studies such as this. We will need to build future versions of the application that make participation very easy and engaging.

We should also highlight that the effects of the C Coach intervention were observed only on NEOFFI Conscientiousness, although the effect on BFI-2 bordered statistical significance (p = 0.053). The implications of this result are not clear at this early stage of intervention development. One might view this critically as the effects due not generalize to other psychological domains. Conversely, one might argue that the treatment effect is specific to the targeted trait or construct. We endeavored to enhance Conscientiousness, and we did not employ behavioral modification interventions for depression or anxiety. In future work, we plan to test the C- Coach intervention in large samples of aged persons, some with normal cognition and others with mild cognitive impairment and look for effects on activities of daily living and biomarkers reflecting physical health.

## Conclusion

The current study provides the first evidence that a smartphone‑based intervention, combined with telecoaching, can enhance Conscientiousness in clinical neurocognitive and aging populations. By empowering individuals to align their behaviors with personal values and goals, it is possible to foster traits that contribute to better health outcomes and quality of life. Implementing such digital‑telehealth protocols in clinical and community settings could offer a novel, scalable approach to supporting resilience, functional independence, and healthy aging. Future research should explore underlying mechanisms, long‑term durability, and AI‑driven personalization to further advance remote personality‑focused interventions.

## Declarations

### Data availability

The dataset generated and analyzed during the current study is not publicly available but may be made available upon reasonable request and approval by the University at Buffalo institutional review board. The underlying code for this study is not publicly available for proprietary reasons.
